# A Revision of the Taxonomy and Identification of *Epipactis greuteri* (Orchidaceae, Neottieae)

**DOI:** 10.3390/plants9060783

**Published:** 2020-06-22

**Authors:** Anna Jakubska-Busse, Elżbieta Żołubak, Marcin Górniak, Zbigniew Łobas, Spyros Tsiftsis, Corina Steiu

**Affiliations:** 1 Department of Botany, Institute of Environmental Biology, University of Wrocław, 50-328 Wrocław, Poland; elzbieta.zolubak@uwr.edu.pl (E.Ż.); zbigniew.lobas@uwr.edu.pl (Z.Ł.); 2 Department of Molecular Evolution, University of Gdańsk, 80-308 Gdańsk, Poland; marcin.gorniak@biol.ug.edu.pl; 3 Department of Forest and Natural Environment Sciences, International Hellenic University, 66100 Drama, Greece; stsiftsis@for.ihu.gr; 4 Association P.P.V.N.C. Excelsior, 310465 Arad, Romania; corinaepipactis@gmail.com

**Keywords:** *Epipactis greuteri*, *E. flaminia*, *E. preinensis*, cpDNA, papillae, ITS, orchids

## Abstract

*Epipactis greuteri* is an obligate autogamous orchid species. Due to large differences in the interpretation of the diagnosis of this species, it is often mistakenly identified by botanists, which results in erroneous information provided in the literature about its distribution in Europe. In the present paper we review its description, including flower details, gynostemium features, and papillae morphology and compare it to *E. helleborine*, with which it is often confused. Based on thorough study of herbarium material (including holotype and isotype) and field research in Greece, Romania, and Poland, we confirm that gynostemium of *E. greuteri* has strongly reduced clinandrium and does not produce viscidium. We also discuss taxonomic treatment of *E. preinensis* and *E. flaminia*, two recently described taxa related to *E. greuteri*. The results of genetic analyses, as well as the range of phenotypic variability of *E. greuteri* individuals from various regions of Europe were presented and discussed. The analysis based on the ITS (internal transcribed spacer) nuclear marker showed no differences among *E. helleborine*, *E. purpurata*, *E. albensis,* and *E. greuteri*, which probably indicates their close relationship. Based on the analysis of plastid regions, six haplotypes were detected in all investigated samples. An exhaustive description of morphological features of *E. greuteri* is provided.

## 1. Introduction

*Epipactis greuteri* H.Baumann & Künkele (Orchidaceae, Neottieae) is a rare species, included in the *Epipactis leptochila* aggregate by Delforge [1]. It was first found in 1972 by G. Hermjakob [2] in a mountainous area in the Pindus range but it was initially identified as *E. leptochila*, a taxon not recorded in Greece till that time. However, the morphological differences between these plants and *E. leptochila,* especially its stable morphological characters, led Baumann & Künkele [3] to describe it as a taxon new to science, giving it the name *E. greuteri*, in honor of the Swiss botanist Werner Rodolfo Greuter. Since that time, *E. greuteri* populations were reported from many localities in different European countries [1,2,3,4,5,6,7,8,9,10,11,12,13,14,15,16,17]. Due to difficulties in species identification, the full range of the phenotypic variability in *E. greuteri* is still unknown. 

It is a species mostly occurring in the Balkan Peninsula, extending to Germany, the Czech Republic, and Poland in the north. So far, it has been found in Greece [1,3,4], Bulgaria [5], Romania [6], Czech Republic [1,7], Germany [1,8,9], Italy [1,10,11], Austria [1,12], Slovakia [13,14], Croatia [15], Slovenia [16], and Poland [17], but in most of these countries the populations are rather small and local.

In Greece, where the species was first described, it forms large colonies and is mainly found along the Pindus range, Mt. Olympus, and the island of Evia, reaching southwards towards the highest mountains of the Peloponnese (Mts. Taigetos, Parnon, and Chelmos) [4]. *Epipactis greuteri* was also noted in the Eastern Alps, from northern Italy to Croatia, as well as south to Émilie-Romagne. Vlčko et al. [14] reported it from Central and Southern Europe, and in Slovakia from Strážovské vrchy, Malá Fatra, Javorníky, Pieniny and Čergov. Localities of this species have also been found in the Polish part of the Western Carpathians [17].

*Epipactis greuteri* was described as an autogamous species [3]. According to the species characteristics published in literature, its anthers are sessile, projecting beyond the rostellum, the clinandrium is poorly developed or absent, the viscidium is inefficient or absent, and it is especially important that the gynostemium structure is analogous to that of *E. muelleri* [1,3,14].

Unfortunately, the features of the gynostemium are rarely taken into account when plants are being determined. Usually, the taxon is identified by botanists in situ mainly on the basis of (a) the elongated pedicels, (b) the pendant and half-opening flowers, and/or (c) the color of the flowers which usually lack pink tones. Due to the controversial features that are the basis for distinguishing infraspecific taxa of *E. greuteri*, determining the true range of the variability of this species is relevant. By reviewing the contradictory literature data, including the original diagnosis, it is still unclear whether the species can form a functional rostellum, clinandrium, and viscidium. The presence of a viscidium is a distinctive characteristic of allogamic species to which *E. greuteri* is not classified. This leads to further complications related to the taxon’s identification and categorization. If it is true that this taxon can produce two types of gynostemium structures (with and without clinandrium and functional viscidium), this means that the column morphology cannot be a basic diagnostic feature of the Helleborines taxonomy.

Apart from the column structure, the size and shape of the conical margin cells (papillae) at the leaf margins have been sometimes used for species identification within *Epipactis* (e.g. [1,18,19,20]). The papillae are usually arranged in three to four rows at the edge of the leaves and along the veins. The usage of micromorphological features of leaves in *Epipactis* taxonomy is however controversial, and data for *Epipactis greuteri* have not been published so far.

In recent decades, the extensive use of DNA markers helped taxonomists to explore the affinities between species within genera or even at higher taxonomic ranks. In such a study, Sramkó et al. [21] used restriction site-associated sequencing (RAD-seq) to explore the phylogenetic affinities of several taxa within the section *Epipactis*. Based on their findings, *Epipactis greuteri,* like many other autogamous species (*E. albensis*, *E. dunensis*, *E. muelleri*, *E. pontica*), originated from local populations of *Epipactis helleborine* s. s. [21]. However, the results of such studies are not always acceptable by taxonomists as there are several concepts of what constitues a “species”.

Here, we mostly focus on the morphological species concept, which is based on gaps in species morphological features. So, due to many mistakes in the determination of both herbarium material and plants identified in situ, in different European countries, and contradictory published data about gynostemium morphology in the species, the aims of the present study were: (i) to determine whether *E. greuteri* develops a functional gynostemium; (ii) to present and discuss the diagnostic characters of *Epipactis greuteri*; (iii) to highlight the differences in the structure of the gynostemium between the autogamous (self-pollinating) *E. greuteri* and the allogamous (cross-pollinated) *E. helleborine*; (iv) to present the range of phenotypical plasticity of *E. greuteri* in reference to its original diagnosis; (v) to discuss the taxonomic treatment of *E. preinensis* and *E. flaminia*, two taxa recently separated from *E. greuteri* and (vi) to examine whether there are genetic differences between *E. greuteri* populations occurring at the edges of its geographical distribution, and other species of the genus *Epipactis*.

## 2. Results

### 2.1. Herbarium Material Investigations—Reexamination of the Holotype and Isotype

**Figure 1:**
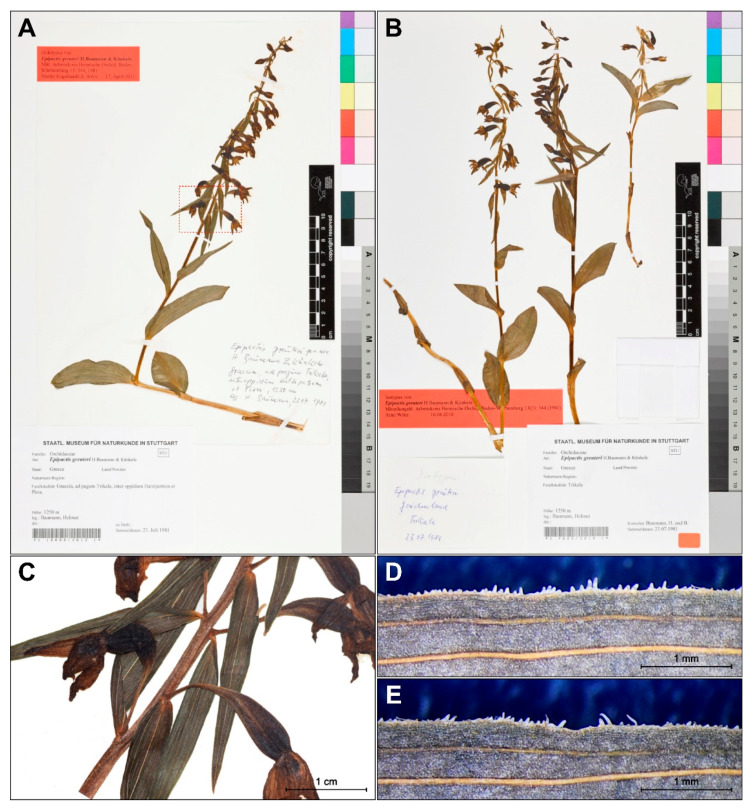
Holotype (STU!, no. 1 10008/2012 1) (**A**,**C**–**E**) and isotypes (STU! no. 1 7645/2016 1) (**B**) of *Epipactis greuteri*; enlargement flowers from the holotype show visible long pedicels and narrow ovary (**C**). The details of papillae arrangement on the leaf margins of the holotype specimens (**D**–**E**).

The holotype of *E. greuteri* contains well preserved plants (STU herbarium specimens nos. 1 10008/2012 1 and 1 7645/2016 1) that were collected on 23 July 1981 by Helmut Baumann in a mountainous area of Trikala (*locus classicus*), a city in northwestern Thessaly, Greece. The type of specimen is represented by one plant with a height of 45.5 cm, with one vestigial and five cauline leaves and also an inflorescence, whose upper flowers are in the bud stage (Figure 1A). Isotypes contain two flowering ramets and one in bud stage (Figure 1B).

Reexamination of both, the holotype and the isotypes, confirms autogamy in *E. greuteri* plants. Importantly, none of the individuals investigated formed a viscidium, or any structure that on the basis of the literature [1] we could name “viscidium inefficient”, which confirms that *E. greuteri* s. str. is a self-pollinated species.

Other important features distinguishing the analyzed species from other Helleborines are the elongated, pendant pedicels (4–12 mm), and the narrow ovaries (Figure 1C). Papillae vary in shape and angle of inclination and are clearly visible on both type specimens.

### 2.2. Field Research—Morphological Analyses

**Figure 2:**
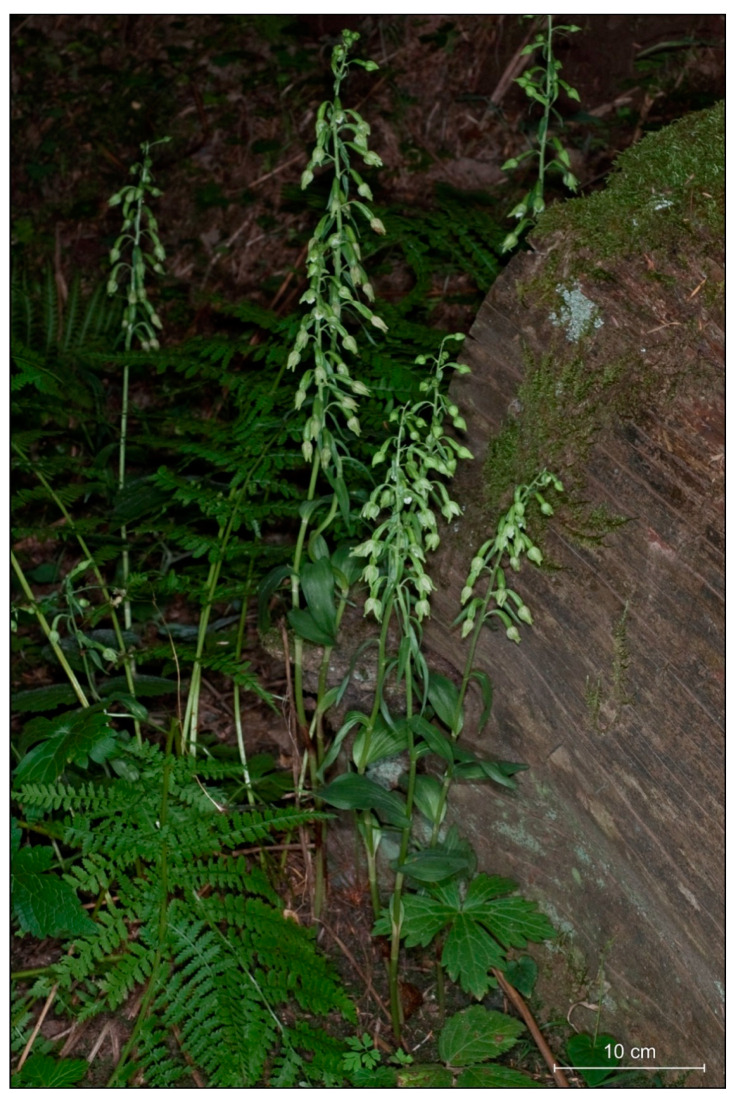
Natural population of *Epipactis greuteri* from Pertouli, Trikala in Greece, close to the *locus classicus*.

**Figure 3:**
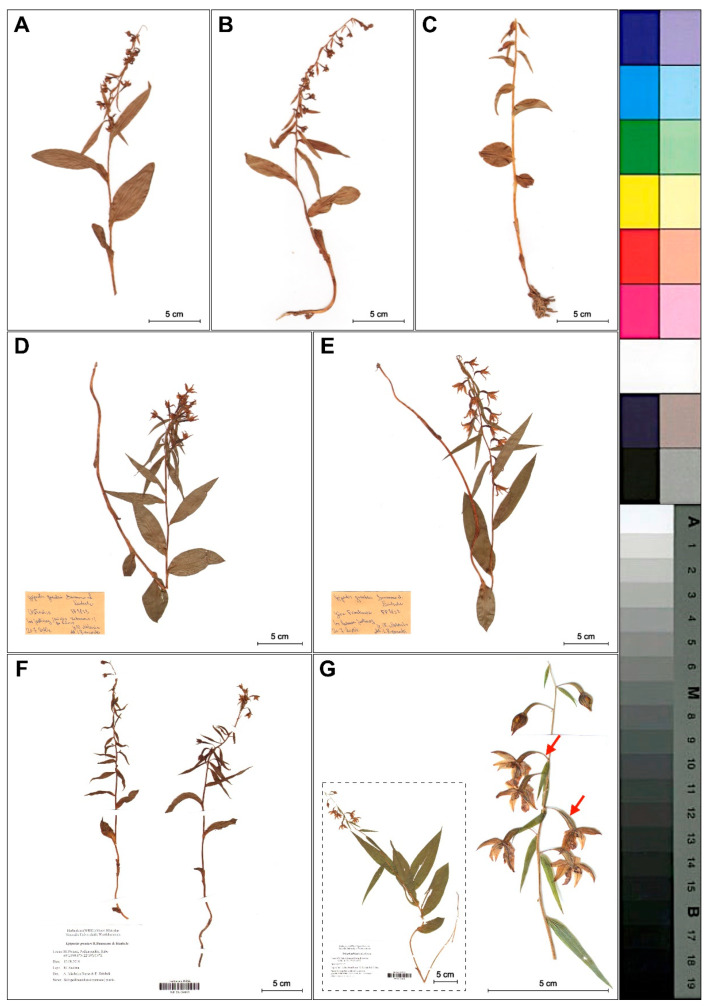
Differences in plant morphology of investigated *Epipactis* taxa. (**A–C**) Dry plants of *E. greuteri* from the Northeastern Carpathians, Semenic Mts., Romania. (**D–E**) Plants with viscidium in flower buds and well developed clinandrium identified by Bernacki as *E. greuteri* from the Przemyskie Foothills, SE Poland. (**D**) Chołowice near Krasiczyn and (**E**) Frankowa Mt. (Maciejówka). (**F**) *E. greuteri* from Rabe, Podkarpackie, SE Poland, (WR GN 064833). (**G**) *E. helleborine* from Kotowice (near Wrocław), SW Poland, (WR SN 064835). The red arrows mark the longer pedicel, narrow ovary and pendant flowers (taxonomically significant features of *E. greuteri*) presented in *E. helleborine* s. str.

We found that *Epipactis greuteri* produces only flowering ramets (Figure 2). Contrary to these rather typical forms of this taxon observed in Greece and Romania, several individuals found in Poland and identified as *E. greuteri* by Bernacki (Figure 3D,E), probably based only on their long pedicels and narrow ovaries, were characterized by a column typical of *E. helleborine* with a functional, well-developed clinandrium and viscidium in the flower buds.

Only two autogamous plants found near Rabe village (Baligród, SE Poland), a new site for Poland, were morphologically similar to the typical individuals of *E. greuteri* (Figure 3F).

**Figure 4:**
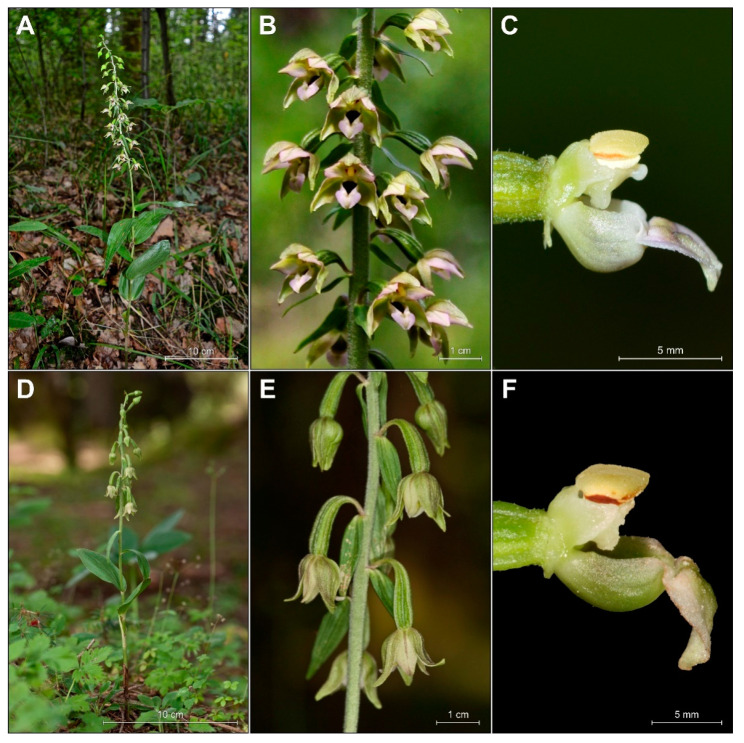
General habit and flower details of the taxa analyzed. (**A**–**C**) *Epipactis helleborine* Poland, Milicz, (**D**–**F**) *E. greuteri* from Greece, (**D**, **E**) Peloponnese, Pertouli-Chrysomilea, and (**F**) Romania; (**C**) and (**F**) details of column morphology: (**C**) *E. helleborine*, (**F**) *E. greuteri*.

However, the analysis of *E. greuteri* individuals from Greece and Romania indicates that the most consistent morphological features of this species were the absence of a viscidium, the slender long ovary with a long pedicel, and associated with this, the pendant flower position, which in most cases are half-opened (Figure 2 and Figure 4D,E).

The plants analyzed were variable in terms of perianth color. In fresh plants, the petal color was nearly pinkish, or pale to olive-green. Determination of flower coloration in herbarium/dried material is difficult because of the material dehydration. In addition, the loss of color and strong flattening of plants during the drying process cause deformation of plant organs, especially flowers, leading often to the erroneous taxon identification. 

**Figure 5:**
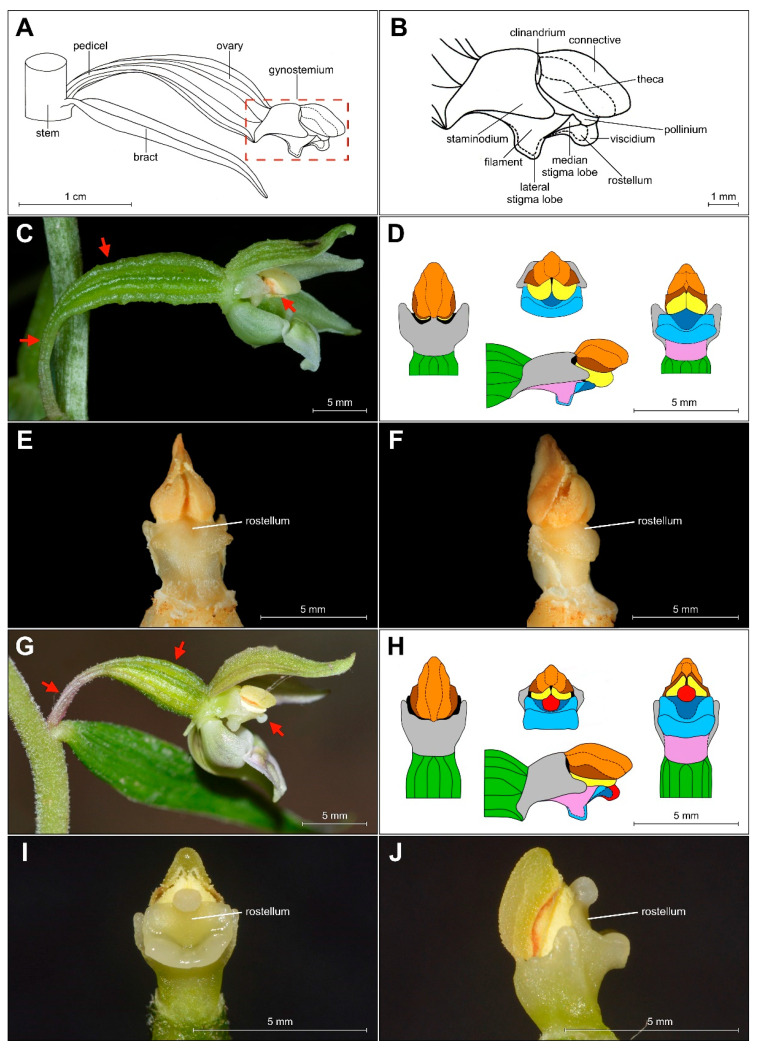
(**A**–**B**) Scheme of *Epipactis* gynostemium, side view. Differences in morphology of gynostemium between *E. greuteri* (material preserved in alcohol) (**C**–**F**) and *E. helleborine* (fresh plant) (**G**–**J**). Various elements of the gynostemium morphology are marked on (**D**) and (**H)** in the different colors: orange—connective, brown—theca, red—viscidium, dark blue—rostellum, light blue—median/lateral stigma lobe, violet/pink—filament, green—ovary, black—clinandrium, yellow—pollinium; red arrows mark important characters: pedicel, ovary, and viscidium (the last presented only in *E. helleborine*).

The shape of the gynostemium is significantly different from that of *E. helleborine*, a common and widespread species of Helleborines in Europe (Figure 5C–F for a comparison between these taxa).

**Figure 6:**
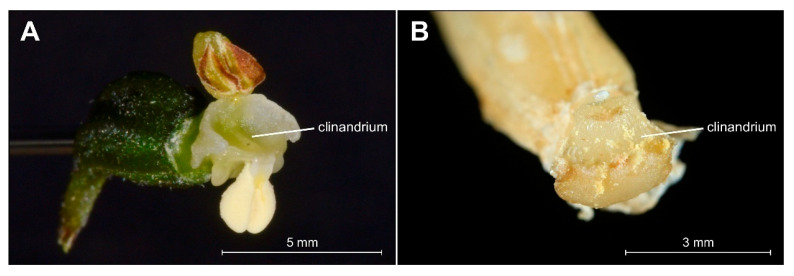
Comparison of details of clinandrium, i.e., the cavity in the upper part of the column that contains the anthers in *Epipactis helleborine* (**A**) and *Epipactis greuteri* (from material preserved in alcohol) (**B**).

The analyzed specimens of *bona fide E. greuteri* were characterized by a unique column structure. Specifically, viscidium was not present and the clinandrium was strongly reduced (Figure 6B).

Data collected on the basis of our field observations [4,6], as well as information from various literature sources (e.g. [1,3,5,6,14,22]) on the characteristics and morphological variability of *E. greuteri* are presented in Table 1.

#### Micromorphology of Papillae

**Figure 7:**
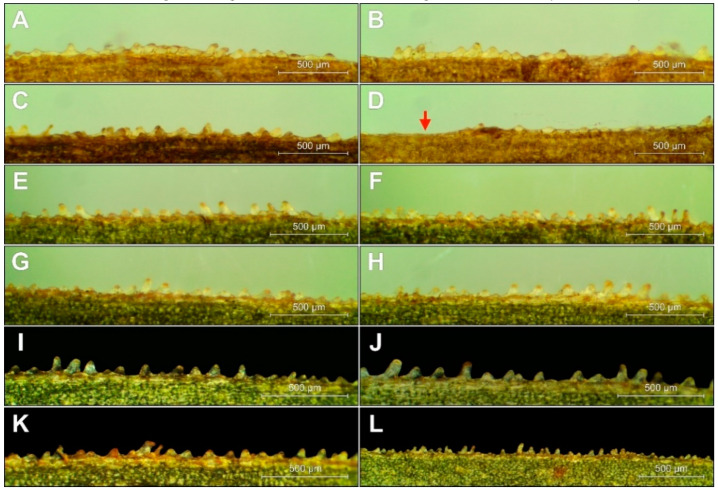
The arrangement of the conical cells (papillae) at *E. greuteri*. Origin of specimens: Romania, Semenic Mts. (**A**–**D**); Greece, Trikala, Pertouli, (**E**–**L**). The red arrow marks without papillae.

The characteristic cone marginal cells, called papillae, were well developed in *E. greuteri*; however, they differed significantly in height and density not only within/between different ramets but also on the same leaf. Similar to other Helleborines, the papillae in *E. greuteri* were arranged in (0-)2–4 rows along the veins and at the edges of the leaves (Figure 7). In general, *E. greuteri* papillae were of different size and shape, vertical or of variable inclination, bending towards the tip of the leaf, forming clusters or appearing separately, even on the same edge of the leaf. The length of the papillae located even along the edges of the same leaf ranges from ca. 20 μm to 183 μm.

### 2.3. Molecular Data

Based on the analysis of plastid regions, six haplotypes were detected in all samples. The variability of the analyzed regions between haplotypes is shown in Table 2. 

**Figure 8:**
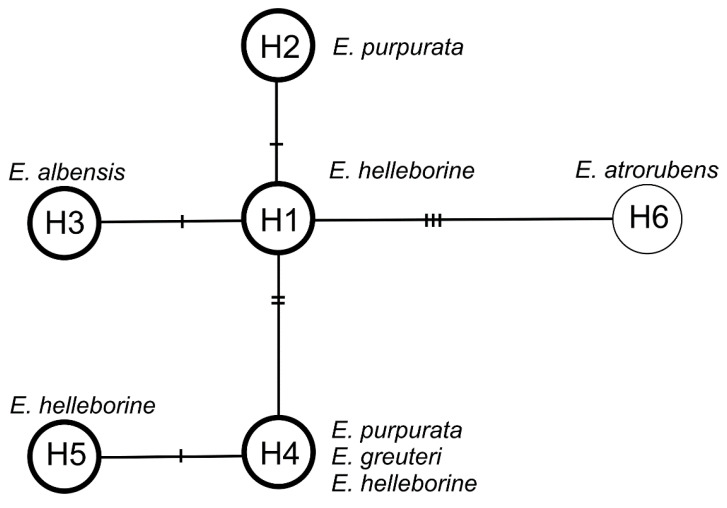
Median-joining network for plastid DNA haplotypes in *Epipactis*. The haplotypes are indicated by circles. The number of mutations required to explain transitions among haplotypes is indicated along the lines connecting the haplotypes by cross hatches. The haplotypes that have identical ITS (internal transcribed spacer) are bold in the circle.

The haplotype network consists of one central (the most common) haplotype H1 connected by one mutation event with haplotypes H2 and H3 and by two mutation events with haplotype H4, which is connected by one mutation with haplotype H5. Haplotype H1 has been reported for *E. helleborine.* Haplotype H2 is characteristic of *E. purpurata,* H3 of *E. albensis*, H4 of *E. helleborine*, *E. purpurata,* and *E. greuteri,* and H5 of *E. helleborine.* Haplotype H6 represent the outgroup species, *E. atrorubens* (Figure 8). On the other hand, the analysis based on the ITS (internal transcribed spacer) nuclear marker showed no differences (the sequences were identical) among *E. helleborine, E. purpurata, E. albensis,* and *E. greuteri*, which probably indicates their close relationship.

## 3. Discussion

### 3.1. Diagnostic Characters in the Protologue of Epipactis greuteri

According to the original Latin diagnosis in protologue of *E. greuteri*, it is characterized by a unique combination of morphological features: (1) rostellum obsolescent, (2) pedicel very elongated, 5–10 mm long, and (3) ovary ca. 20 mm long.

In the detailed description by Baumann and Künkele [3], the authors state that “rostellum is only present and functional at the stage of buds or in freshly opened flowers”. However, such a statement about its morphology could cause misinterpretation and incorrect identifications of plants.

According to Darwin’s definition, the rostellum is strictly a single organ, formed by the modification of the dorsal stigma and the pistil [23]. In the botanical literature, the term is treated in two ways: broadly, it is a transformed median stigma lobe that differs at least morphologically from fertile lobes [24,25,26,27] and precisely, it is only the part of the middle stigma lobe [28,29,30,31,32,33,34]. Rostellum produces structures such as the viscidium, a recurved apex named hamulus, and a dorsal cuticle called a tegula. The function of the rostellum is to separate the anther from the stigma and, by producing the viscidium, to make pollen masses stick on the body of visiting and pollinating insects.

In view of the above, it seems that Bauman and Künkele [3] in the protologue for *E. greuteri* should have rather indicated the absence of a viscidium, as an important character, not the “rostellum obsolescent”. This is confirmed also by the analysis of the holotype flower, as well as the results of our morphological investigations (Figure 1, Figure 5, and Figure 6).

According to the characteristics of the *species nova* given by Baumann and Künkele [3], plants of *E. greuteri* are similar to *E. leptochila*. However, given the wide range of morphological variation observed in Helleborines, it seems that the only similarity between those two species is the absence of a viscidium. This issue definitely requires detailed clarification. Interestingly, in some references (i.e., [1,14]), it is stated that *E. greuteri* produces a column like the that of *E. muelleri*. This is not contradictory because both species (i.e., *E. leptochila* and *E. muelleri*) are autogamous. Autogamy is related to the unique structure of gynostemium that characterizes all the self-pollinated *Epipactis* species.

Based on the examined material, we confirmed that *E. greuteri* does not produce a viscidium (Figure 5C–F), as it is not possible for obligate autogamous *Epipactis* species to have such a floral structure. This is also confirmed by our research on the development of gynostemium of other autogamous species found in Europe (e.g., *E. albensis*) (unpublished data). However, it is mentioned in the literature and it has been also noticed by the authors that viscidium can be absent even in allogamous species, as it withers in dry weather conditions or it can be removed by visiting insects [35].

The term “viscidium inefficient” appearing in the literature in reference to *E. greuteri* (e.g., [1]) is also very vague. Similarly, the feature “rostellum with non-functional viscidium” used in species characteristics (e.g., by Szeląg et al. [17]) results in errors in species identification.

Another floral structure that is commonly used in *Epipactis* taxonomic issues is the clinandrium. Based on the definition of this term, clinandrium is a cavity in the upper part of the column of an orchid flower that contains the anthers (e.g., [23]). According to some authors [1], the clinandrium of *E. greuteri* is “poorly developed or almost absent”. This is in accordance with the results of our research. Specifically, the examination of both the holotype and isotypes, as well as fresh plants and herbarium specimens from Greece and Romania showed that clinandrium in *E. greuteri* is present but strongly reduced compared to allogamic species (Figure 4F and Figure 5C–F).

### 3.2. The Controversy on the Taxonomic Position of the Epipactis greuteri Infraspecific Taxa

Problems with the identification and taxonomic treatment of this species are also confirmed by the description of *Epipactis greuteri* subsp. *flaminia* (P. R. Savelli & Aless.) H. Baumann, Künkele & R. Lorenz (syn. *Epipactis greuteri* var. *flaminia* (P. R. Savelli & Aless.) Kreutz) from Italy. This taxon is currently considered as a separate species—*Epipactis flaminia* P. R. Savelli & Aless. According to Savelli and Alessandrini [36], it differs from *E. greuteri* “mainly because of the complete absence of the rostellum”. Delforge [1] reports that this taxon has a “column lacking a clinandrium”, whereas according to Baumann et al. [22] morph ‘*flaminia*’ produces a strongly reduced clinandrium and does not produce a viscidium. Based on these rather contradictory descriptions, it might be hypothesized that Savelli and Alessandrini in their initial description observed a dry or greatly reduced clinandrium and rostellum, whereas based on the description provided by Baumann et al. [22] ‘*flaminia*’ cannot be delimitated from typical *E. greuteri* individuals, as in the latter species clinandrium is present, but strongly reduced in size (Figure 5).

Another controversial taxon that was described as an endemic to the Rax massif in Lower Austria and is considered as a separate species is *Epipactis preinensis* (Seiser) Landolt, which was formerly named as *Epipactis greuteri* var. *preinensis* (Seiser) P.Delforge or *Epipactis greuteri* subsp. *preinensis* Seiser [37]. According to Seiser’s original description, this taxon is different from *E. greuteri* by the following combination of features: “ratio of leaf-length and length of the internode, shorter pedicels, completely lacking rostellum, wide opened flowers and growing often in clusters” [37]. The terminology used above is also indefinite and insufficient to delimitate both taxa. Specifically, the lack of a rostellum might mean that it is strongly reduced, as in the case of *E. greuteri* (Figure 5). In general, the diagnosis problem is a result of differences in the interpretation of the naming of the part of gynostemium between different botanists, and also applies to the use of diverse terminology to the same morphological elements. Unfortunately, this is rather a general problem in the species and groups of species classification, within the genus *Epipactis*. There is not any widely accepted terminology so far, and there are problems and differences among botanists when treating, delimiting, and identifying *Epipactis* taxa. On the other hand, according to Baumann et al. [22], *E. greuteri* var. *preinensis* produces more open, less hanging and “more colorful flowers” (usually lightly washed violet) and produces a viscidium. In general, species that have the viscidium are also characterized by a well-developed rostellum, a fact that contradicts the original description of *E. greuteri*. As a result, it is possible that it is rather a hybrid of *E. greuteri* with *E. purpurata* (syn. *E. viridiflora*) as Baumann et al. [22] suggests. Another possible hypothesis is that this is a hybrid with another Helleborine species, or it is an incorrectly/erroneously described taxon, requiring revision. The identification of *E. greuteri* var. *preinensis* is difficult and problematic because other authors (e.g., Delforge [1]), described this as a taxon “always lacking viscidium”. The taxonomic status of this taxon is still unclear as it is possible that it is only a manifestation of the wide range of *E. greuteri* phenotypic plasticity. An intricate taxonomic treatment of *E. greuteri* is presented in Table 3.

### 3.3. Species Variability in Taxonomic Context

Although, an exhaustive description of *E. greuteri* including detailed information about the color of the perianth is included in the protologue, this issue still raises doubts among botanists. Many researchers argue that *E. greuteri* has only green flowers (usually lacking pink tones) or, on the contrary, only pale pink [1]. This raises far-reaching controversy, because when individuals have features compatible with the protologue, but their flowers are green, they are identified as a separate taxonomic unit (i.e., *E. greuteri* subsp. *flaminia*).

In the original Latin description of the species it is clearly defined that *E. greuteri* is a species whose perianth color can be variable: “(…) Sepala ovato-acuta, 9–12 mm longa et 4–5 mm lata, viridia vel rosea; petala ovata, 8–9 mm longa et 4–5 mm lata, rosea. (…) Epichilum (…), albidum vel viridium vel roseum, (…); hypochilium (…), intus fusco purpureum vel flavo viridium” [3]. Our field studies on populations located at different geographic locations with large distances between them (e.g., Greece and Poland), and also online data [20] confirm that the color of the flowers can vary from whitish-green to some tint of very light pink in the epichile and on the edges of the tepals. The size and shape of the epichile can vary as well; for example, in some populations it is significantly wider as it is in the Southern Carpathians, in others it is narrower or longer than the hypochile, as it is in the Southwestern Carpathians, in the Semenic Mountains.

In view of the above, it should be considered that the color and shape of the perianth in this species may be variable and cannot be the basis for the description of additional taxonomic units (subspecies or varietas).

*Epipactis greuteri* is often known to be less variable within populations, but important detailed morphological differences can be observed between different populations, especially if they are located at a large geographical distance. In fact, this may be the cause of erroneous identifications, because *E. greuteri* grows in areas where *E. helleborine* may also be present. Specimens from the population in the SW Carpathians (the Semenic Mts., W Romania), produce leaves that are placed high above the ground, smaller or shorter than the respective internodes. The same variation associated with leaf shapes can be seen in another population located in central Romania, in the Southern Carpathians in the Bucegi Mts. 

Between different populations analyzed in Romania we observed morphological differences at the gynostemium. Individuals of *E. greuteri* from the Bucegi Mts. have a more reduced clinandrium than the exemplars from the Semenic Mts. In both cases the viscidium was not present. The pollinia were crumbled and spread over the stigmatic surface.

**Figure 9:**
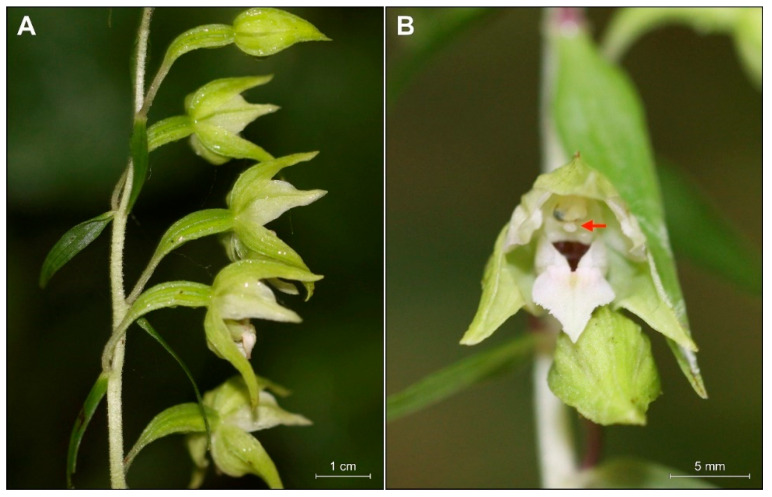
Phenotypical variability of *Epipactis helleborine* (L.) Crantz. Locality: Siechnice near Wrocław, SW Poland. (**A**) Side view of the inflorescence, please pay attention to long pedicels and narrow ovaries. (**B**) Flower, frontal view. Viscidium is marked with a red arrow. This specimen was prepared as herbarium vouchers presented in Figure 3G.

Based on the material examined in the present study, we can state that all *E. greuteri* individuals were autogamous. The fact that specific authors [22] regard *E. greuteri* from Calabria, in Italy, as a species that can form a functional viscidium, might indicate misidentification of other *Epipactis* taxa or local adaptations. It is worth mentioning that the absence of the viscidium has also be noticed in *E. helleborine* plants, but this definitely is owned to visiting insects. As a result, such ramets are often misidentified as *E. greuteri*, from which it differs in the morphology of gynostemium (Figure 4, Figure 5 and Figure 6). These mistakes seem to be the result of species identification based solely on one specific feature, namely, elongated pedicels on which flowers are hanging. As indicated by the results of our field research and analyses of herbarium material, the presence of long pedicels is also a feature of some *E. helleborine* individuals that grow under light deficiency and/or in acid soils (Figure 3G and Figure 9). Unfortunately, such forms of *E. helleborine* are also identified by some researchers as a hybrid of *E. helleborine* and *E. greuteri*, named *Epipactis* × *breinerorum* [41].

The size, shape, and arrangement of the papillae at the leaf margins of *E. greuteri* were very variable, and thus they cannot be used for taxonomic identification. This was also confirmed by the results of earlier studies carried out by Jakubska-Busse and Gola [19] on other *Epipactis* species.

Care should also be taken when distinguishing *E. greuteri* only on the basis of the habitat in which the taxon occurs. This species grows in shady sites, on deep moisture calcareous soils, such as in montane coniferous forest communities (*Galio-Abietenion*), beech forests (*Eu-Fagenion*), forests of slopes, screes and ravines (*Tilio-Acerion*), acidophilic montane beech forest (*Luzulo-Fagenion*), and/or in riparian alder forests (*Alnenion glutinoso-incanae*) [42]. The populations of *E. greuteri* from Semenic Mts. and Bucegi Mts. in Romania, although located at a great geographical distance, have the same habitat preferences, preferring locations with a deep, moist substrate and dense shade, generally a low vegetation carpet and forests dominated by *Picea abies, Abies alba,* and *Fagus sylvatica* [6]. In Greece, *E. greuteri* is mostly found in *Abies borisii-regis* and *A. cephalonica* forests and in *Abies-Fagus* mixed forest, whereas a few scattered and isolated individuals have been found in *Fagus sylvatica* forest [4]. Moreover, it has been recorded once under *Platanus orientalis* individuals occurring along a small stream [4].

### 3.4. Molecular Data

Studies carried out by Madesis et al. [43] using the DNA barcoding regions rbcL, matK, and ITS-2 showed no difference between *E. helleborine, E. greuteri,* and *E. purpurata* when using the DNA barcoding regions rbcL and ITS-2, whereas *E. purpurata* differed from *E. helleborine* in matK. Recent studies carried out by Sramkó et al. [21] clearly showed that there is an ancestral variability displayed by *Epipactis helleborine* s.s. that forms a basis for the derivative species including *E. greuteri* and other autogamous species of the genus *Epipactis*. Our results, on the basis of both plastid and nuclear DNA analyses, confirm that conclusion. The occurrence of other ancestral plastid haplotype was also suggested by Tranchida-Lombardo et al. [44] for other species within the genus *Epipactis*. The results of the present study show variability of plastid haplotypes. Considering the results of the analysis of Sramkó et al. [21], the occurrence of the same haplotypes in different species (*E. helleborine, E. greuteri,* and *E. purpurata*) is a result of incomplete lineage sorting and does not reflect phylogenetic relationships. By interpreting the generated network (Figure 8) in the context of the results obtained by Sramkó et al. [21], we also confirm the basal position of *E. purpurata* in relation to *E. helleborine* s. s. and many autogamous species, as two of four haplotypes that exist in *E. helleborine* s. s. were found in *E. purpurata*. However, we cannot agree with Sramkó et al. [21] and classify many autogamous species (*E. greuteri, E. pontica, E. muelleri, E. albensis,* and *E. dunensis*—clades G, H, I, J, and K respectively on the phylogenetic tree) within *E. helleborine* s. l., in order to avoid paraphyly in *E*. *helleborine* s. s.

The problem of the actual distribution of this taxon in Europe remains unsolved. Due to large differences in the interpretation of the *E. greuteri* diagnosis, this species is often mistakenly identified by botanists, which results in erroneous information provided in the literature about its occurrence and range. In our opinion, it is necessary to expand this study by exploring the morphological variability of populations from the entire range of its distribution, especially in Central Europe.

## 4. Materials and Methods

### 4.1. Herbarium Material Investigations

The holotype and isotype of *Epipactis greuteri* were re-investigated. In addition to the holotype and isotypes, dry plants and herbarium specimens collected from Bulgaria, Czech Republic, Greece, Poland, Romania, and Slovenia were also examined and used in this study. The investigated herbarium sheets are listed in the Appendix A.

### 4.2. Field Research

Plant material from natural populations of *Epipactis greuteri* in Greece, Pertouli, the region of Trikala (close to the *locus classicus*), Romania (Semenic and Bucegi Mts.), and SE Poland (Western Carpathians Mts., Baligród Forest District, Rabe) were investigated and compared with *E. helleborine* s. str. GPS (Global Positioning System) coordinates are available from the authors upon request.

### 4.3. Microscopic Analyses of the Papillae Morphology

Dry leaves collected from natural populations of *Epipactis greuteri* from Greece, Romania, and Poland were analyzed with the use of microscopic techniques according to the methodology of Jakubska-Busse and Gola [19]. The observations and documentation of the conical marginal cells (papillae) on the leaves margins, were performed with the use of a Nikon Eclipse 600 optical stereo-microscope (Nikon Instruments, Europe B.V.), an Olympus BX-50 microscope, and a DP71 camera system supported by Cell^B software (Olympus, Olympus Optical Co.). The software ImageJ (Wayne Rasband, NIH, USA) was used to measure the length and density of papillae.

### 4.4. Molecular Analyses

Plant material was sampled from fifteen specimens of investigated natural populations of *E. greuteri*: ten samples from Greece (Pertouli), three samples from Romania (Semenic Mts., Bârzava valley), two samples from Poland (village Rabe, near Baligród).

In the genetic variability analyses the nuclear ribosomal DNA (ITS1-5.8S-ITS2), intron *rpL16,* and two plastid spacers *matK-5’trnK^(UUU)^* and *trnS^(GCU)^*-*trnG^(UCC)^*-*trnG^(UCC)^* were used. Sequences were deposited in GenBank (Accession Nos. MN848517 to MN848523, MN850336-MN850339 and MT312214-MT312219). Total genomic DNA was extracted from 20 mg of silica-dried leaves [45] using a Genomic Mini AX Plant (A&A Biotechnology, Gdynia, Poland). To homogenize the samples, Lysing Matrix A and FastPrep (MP Biomedicals, USA) were used. Biometra T Gradient and Eppendorf Mastercycler machines were used to amplify all regions. ITS was amplified using two sets of primers (17SE, 26SE; [46]). The *rpL16* intron was amplified using rpL16F71 and rpL16R1516 primers [47]. For *matK-5’trnK*, we employed matK5’R and matK6 primers [48]. The region including the *trnS-trnG* intergenic spacer and the *trnG* intron was amplified using trnS^(GCU)^ and trnG^(UCC)^ primers [49]. Polymerase chain reaction (PCR) amplifications were carried out in a total volume of 25 µL, containing 2.5 µL of 10 × buffer, 1 µL of 50 mM MgCl2, 1 µL of 5 mM deoxynucleoside triphosphates (dNTPs), 1 mL of 10 mM of each primer, 1 mL of dimethyl sulfoxide (DMSO) (only ITS), and 0.5 µL of 2.0 unit TaqNova DNA polymerase RP710 (Blirt-DNA Gdańsk, Poland). The PCR conditions for all the regions are described in Table 4. PCR products were purified using a Wizard PCR Preps DNA Purification System (Promega, Madison, WI, USA). Tubes containing 5 μL of purified PCR product and 5 μL of 5 μM primer (the same as used for PCR amplification) were sent to Macrogen (Amsterdam, Netherlands) for sequencing. Using FinchTV and SeaView programs, obtained sequences of *E. greuteri* and *E. helleborine* were identified and compared with (downloaded from NCBI nucleotides databases, respectively *rpl16*, *matK-5’trnK*, *trnS-trnG-trnG*) the following taxa: autogamous *E. albensis* (JN811743, JN811758, JN81817), and also allogamous, *E. helleborine* (JN811747, JN811759, JN811806), *E. purpurata* (JN811757, JN811771, JN811815, JN811749, JN811766, JN811811), and outgroup taxon *E. atrorubens* (JN811744, JN811772, JN811820). Relationships among plastid haplotypes were analyzed in the PopArt software (v. 1.7) using median-joining networks [50].

## 5. Conclusions

According to the protologue, *Epipactis greuteri* is an autogamous taxon with a gynostemium similar to that of *E. leptochila*. Neither species produces a viscidium. Identification of *Epipactis* species, especially the self-pollinated ones, should include a detailed examination of the gynostemium structure. The determination of *Epipactis* with long pedicels, narrow ovaries, and hanging flowers, which at the same time have well-developed clinandrium and viscidium as in *E. greuteri* should be considered carefully. A well-developed clinandrium and viscidium do not correspond to the original diagnosis of the species, as well as to the holotype and isotype, and as a result, any identification of such an *Epipactis* individual as *E. greuteri* would be incorrect. Further research is certainly needed, using both extensive measurements of morphological features and DNA analyses with samples across the whole range of species distributions, which will help to adequately explore the relationships between the taxa within section *Epipactis*.

## Figures and Tables

**Table 1:** Morphological features of *Epipactis greuteri*.

Characters	Variable Type
Stem number	1 or a few clustered
Stem height (cm)	(12‒)20‒60(‒76)
Stem color	Dark green, greenish-grey, greenish-violet, sometimes washed violet at base
Stem indumentum	Dense, whitish pubescence
Leaves color	Dark green
Cauline leaves number	4–8
Bract length (long/width, cm)	(2‒)5.5 × (2‒)3.5‒5.5(‒7.5)
Upper leaf size (cm)	4‒6 × 2‒4.5
Median leaf sizes (cm)	3.5‒11 × 2‒6.5
Lower leaf size (cm)	2‒5 × 1.3‒3.5
Superior cauline leaf aspect	Bract-like
Median leaf shape	Lanceolate, spreading-erect, narrow, arching; lower 1–3 leaves short and rounded
Leaf margin edge	Regularly serrated, papillae of different height and length
Number of flowers	5‒25(‒30)
Inflorescence length (cm)	Up to 18 cm
Inflorescence aspect	Elongated, occupying up to half the stem
Flower aspect	± opening, bell-shaped, pendant
Sepal size (mm)	9‒12 × 4‒5
Sepal color	Pale green, greenish with a pink tint and apex bent backwards, pale pink
Sepal shape	Oval-acuminate, keeled
Petal size (mm)	Near equal as sepal
Petal color	Whitish with the center and apex yellowish-green or seldom light pink
Hypochile size (mm)	4‒5 × 4‒5
Hypochile color outside	Greenish-white
Hypochile color inside	Yellowish-green, olive green to reddish-brown and shiny
Hypochile aspect	Nectariferous
Hypochile-epichil connection	“U”-shaped
Epichile size (mm)	3‒4.5 × 4‒5
Epichile color	White, greenish or pinkish
Epichile bosses	2 attenuated
Labellum size/aspect	7‒9 mm long, × 4‒5 mm, margins sometimes undulate
Ovary size (mm)	6‒15 × 3‒6
Ovary shape	Peer-shaped, narrow
Ovary color	Yellowish-green,
Ovary indumentum	Glabrescent
Ovary pedicel length	Very elongated, (4‒)4.5–12 mm
Ovary pedicel color	Green at base, washed yellowish
Gynostemium size (mm)	Ca. 3.5‒5.5 × 1.5‒3.5
**Viscidium**	**Absent**
Anther	Sessile, projecting beyond rostellum
Rostellum	Presence
**Clinandrium**	**Strongly reduced**
Stigma	Orientated downwards or towards the anther
Pollinia Flowering period	Friable and then crumbling, disintegrating onto the stigma Late June to early September

**Table 2:** Alignment of plastid haplotypes of all analyzed taxa and regions. ‘+’ indicates fragment duplication.

Haplotypes	*trnS-trnG*				*5’trnK-matK*					*rpl16*	Specimens
	47	311–324	486	519	193	395	483	562	948	366–375	
**H6**	T	-	C	A9	C	G	A9	T8	C	-	*E. atrorubens*
H5	G	+A	A	A10	C	A	A9	T11	C	+	*E. helleborine*
H4	G	+C	A	A10	C	A	A9	T12	C	+	*E. helleborine* *E. purpurata* *E. greuteri*
H3	G	-	A	A9	C	A	A11	T10	T	-	*E. albensis*
H2	G	-	A	A9	A	A	A11	T11	C	-	*E. purpurata*
H1	G	-	A	A9	C	A	A11	T11	C	-	*E. helleborine*

**Table 3:** Taxonomic treatment of *Epipactis greuteri*, according to Delforge [1] and online databases [38,39].

**Classification of *Epipactis greuteri***		
A species complex (aggregate) (distinguished only by Delforge [1])	*Epipactis leptochila* agg.	
Species	*Epipactis greuteri* H.Baumann & Künkele	
**Nomenclature Investigation**		
**Infraspecific Taxa Previously Classified to *E. greuteri***	**Synonyms**	**Current Accepted Names**
*Epipactis greuteri* subsp. *preinensis* Seiser	*Epipactis preinensis* (Seiser) Landolt	*Epipactis flaminia* P.R.Savelli & Aless.*
*Epipactis greuteri* var. *preinensis* (Seiser) P.Delforge		
*Epipactis greuteri* subsp. *flaminia* (P.R.Savelli & Aless.) H.Baumann, Künkele & R.Lorenz	–	*Epipactis flaminia* P.R.Savelli & Aless.
*Epipactis greuteri* var. *flaminia* (P.R.Savelli & Aless.) Kreutz	–	
*Epipactis greuteri* var. *aspromontana* (Bartolo, Pulv. & Robatsch) P.Delforge	*Epipactis aspromontana* Bartolo, Pulv. & Robatsch *Epipactis helleborine* subsp. *aspromontana* (Bartolo, Pulv. & Robatsch) H.Baumann & R.Lorenz	*Epipactis leptochila* subsp. *aspromontana* (Bartolo, Pulv. & Robatsch) Kreutz
*Epipactis greuteri* subsp. *sancti-bruni* Bongiorni, De Vivo, Fori & Pisani	–	*Epipactis greuteri* H.Baumann & Künkele

* According to Průša [40], the name *E. flaminia* is a synonym of *E. greuteri*.

**Table 4:** Polymerase chain reaction (PCR) conditions for amplified regions.

Amplified Region	Initial Denaturation, °C/time	Denaturation, °C/time	Annealing, °C/time	Elongation, °C/time	Final Elongation, °C/time	No. of Cycles
ITS	94/3 min	94/45 s	52/45 s	72/60 s	72/5 min	29
*rpL16*	80/5 min	95/60 s	50/60 s	65/4 min	65/7 min	30
*matK-5’trnk*	80/5 min	94/60 s	50/60 s	75/2 min	72/7 min	28
*trnS-trnG-trnG*	80/5 min	95/60 s	50/60 s	65/4 min	65/7 min	30

## Appendix A

The list of investigated herbarium material of *Epipactis greuteri*, according to the label data.

**Bulgaria.** The Rhodope Mts. (Central): along the road from Teshel to Mugla village, at about 10 km of Teshel, in a beech forest, KG-91, with fruits, 24.07.1998 *A.S. Petrova, D. Venkova & I. Gerasimova* (SOM 155338). Czech Republic. Flora moravica. 75. Jesenické podhůří, 6169d, Paseka (distr. Olomouc), část Karlov, na pravém břehu potoka Tepličky ve fragmentu jedlobučiny 1,7 km V od kapličky v Karlově, 26 exempl, 494900,8 N, 171605,0 E, 425 m a.s.l., 02.08.2015 *P. Batoušek s.n.* (BRNU 643185); Mor. kras. Rudické propadání při hranici v nejjižnějším cípu NPP 07.09.2002 *J. Unar s.n.* (BRNU 649530). **Greece.** Graecia, ad pagum Trikala, inter oppidum Hatzipetrion et Pirra, 1250 m a.s.l., 23.07.1921 *H. Baumann s.n.* (holotype: STU 1 10008/2012 1); Trikala, 1250 m a.s.l., 23.07.1981 *H. Baumann s.n.* (isotype: STU 1 7645/2016 1). **Poland.** The Beskid Sądecki Mts., the Radziejowa Range, Jaworzynka stream valley near Gołkowice Górne, ca. 580 m a.s.l., ATPOL EG2441, along forest road, 30.07.2008 *K. Stawowczyk s.n.* (KRA 0385069); the Beskid Sądecki Mts., the Radziejowa Range, Jaworzynka stream valley near Gołkowice Górne, ca. 710 m a.s.l., ATPOL EG2441, over stream along forest road, 30.07.2008 *K. Stawowczyk s.n.* (KRA 0385072); the Beskid Sądecki Mts., the Radziejowa Range, Roztoka Mała stream valley, ca. 630 m a.s.l., ATPOL EG3414, *Petasites albus* community along stream, 01.08.2007 *K. Stawowczyk s.n.* (KRA 0385071); the Beskid Sądecki Mts., the Radziejowa Range, behind the Bachnaty stream valley near Gaboń, *Abies alba* forest along stream, 650 m a.s.l., ATPOL EG2430, a few flowering plants observed, 25.07.2008 *K. Stawowczyk s.n.* (KRA 0385070); the Przemyskie Foothils, Frankowa Mt. (Maciejówka) hill near Bircza, 440 m a.s.l., in *Fagus sylvatica*-*Abies alba* forest, 20.07.2010 *M. Wolanin s.n.* (herb. of Department of Botany, Rzeszów University); the Przemyskie Foothils, 1 km S of Chołowice near Krasiczyn, 250 m a.s.l., *Abies alba* forest, 3 flowering plants, 21.07.2010 *M. Wolanin s.n.* (herb. of Department of Botany, Rzeszów University); Podkarpackie, Rabe, 49°21′49.0″N 22°39′57.9″E, self-pollinated (autogamous) plants, 12.08.2019 *M. Scelina s.n.* (WR GN 064833). **Romania.** The Semenic Mts., Barzava valley, mixed coniferous-beach forest, 45.140549 N, 21.980946 E, 700m a.s.l., ca. 15 ramets observed, 30.07.2010 *C. Ardelean s.n.* (3 plants in A. Jakubska-Busse collection, Department of Botany, University of Wrocław). **Slovenia.** Slowenien, S Laibach, Kocecvje (Rog Baza), Buchenwald; ca. 550m a.s.l., 01.08.1987 *L. Freidinger s.n.* (GJO 0001039, GJO 0001040).
